# The Use of Recycled Cement-Bonded Particle Board Waste in the Development of Lightweight Biocomposites

**DOI:** 10.3390/ma17235890

**Published:** 2024-12-01

**Authors:** Girts Bumanis, Pauls P. Argalis, Maris Sinka, Aleksandrs Korjakins, Diana Bajare

**Affiliations:** Institute of Sustainable Building Materials and Engineering Systems, Faculty of Civil and Mechanical Engineering, Riga Technical University, Kipsalas Str. 6A, LV-1048 Riga, Latvia; girts.bumanis@rtu.lv (G.B.); pauls-pavils.argalis@rtu.lv (P.P.A.); maris.sinka@rtu.lv (M.S.); diana.bajare@rtu.lv (D.B.)

**Keywords:** cement-bonded particle boards, wood wool, recycling, biocomposites, drying, waste management, sustainable construction

## Abstract

Cement-bonded particle boards are gaining popularity globally due to their durability, strength, and, more importantly, environmental sustainability. The increasing demand for these materials has also created the necessity for the sustainable recycling of these materials. In this study, the potential to recycle wood-wool cement board (WWCB) waste into new lightweight insulation biocomposite material was examined. The waste WWCBs were crushed and separated into a fine aggregate fraction, and WWCB production line residues were also collected and compared. The crushed WWCBs were used to produce biocomposites with various compaction ratios and different binder-to-aggregate ratios. To improve their thermal properties and reduce their density, hemp shives were used to partially replace the recycled WWCB aggregate. Their physical, mechanical (compressive and flexural strength), and thermal properties were evaluated, and the drying process of the biocomposites was characterized. The results showed that the density of the produced biocomposites ranged from 390 to 510 kg/m^3^. The reduction in density was limited due to the presence of cement particles in the aggregate. The incorporation of hemp shives allowed us to reduce the density below 200 kg/m^3^. The thermal conductivity of the biocomposites ranged from 0.054 to 0.084 W/(mK), placing the material within the effective range of natural biocomposites. This research has demonstrated that industrially produced WWCBs can be successfully recycled to produce sustainable lightweight cement-bonded insulation materials.

## 1. Introduction

The market for cement-bonded lignocellulosic particle boards has experienced steady growth, driven by various factors, such as increasing awareness about sustainable construction practices, rising demand for eco-friendly building materials, and advancements in manufacturing technology. The market size of global cement-bonded particle board was valued at USD 1.76 Bn in 2022 and is expected to exhibit a compound annual growth rate of 4.7% from 2023 to 2030 [1]. Based on product type, the cement-bonded particle board market is segmented into fiber cement boards, wood-wool cement boards (WWCBs), wood-strand cement boards, and cement-bonded particle boards. WWCBs have a share of 28% in the market for global cement-bonded boards [2]. WWCBs are a versatile building material made from wood-wool shavings (excelsior) and cement. The worldwide acceptance of WWCBs proves their versatility in applications and their durability in any climatic condition. WWCBs are not vulnerable to attack by termites or vermin. Additionally, they are not subject to biological decay.

Moreover, they are classified under both the standard B-s1,d0 class and the higher A2-s1,d0 class (non-combustible), as per EN 13168 and EN 13964 (for acoustic ceiling panels) [3,4]. WWCBs are a wood-based product manufactured from renewable resources, meaning fewer embodied carbon emissions. They are lightweight, easy to process and fabricate, and available to the construction industry in place of less eco-friendly materials, such as common brick or other masonry elements. Panels can be categorized as low-density materials, with a density in the range of 300 to 500 kg/m^3^ [5]. The typical preparation of WWCBs consists of wood wool, cement, and water at a ratio of 2:1:1 (by weight), which are mixed until all the wood wool is thoroughly coated with the cement paste. Previous research results show that the mechanical strength of WWCBs was greatly influenced by the density of the boards [6]. The main problem when producing cement-bonded wood composites is the incompatibility between cement and wood, which may affect the hydration; thus, the final strength of the composites is sensitive to wood extractives [7]. Research on WWCBs continues, as additional supplementary cementitious materials are often incorporated into the mixture’s composition. An industrial by-product, such as paper sludge ash, with good pozzolanic properties and without any hazardous contaminants, is created and integrated as a replacement for cement in WWCBs. Boards are produced by applying a mixture of paper sludge ash and cement with a water-to-powder ratio by mass of 1.0 and a wood-to-powder ratio by mass of 0.75 [8].

Another use of WWCBs has been proposed by entrepreneurs. They have indicated that WWCBs can serve as an integral part of hempcrete building assemblies in the following ways [9]:

- As a permanent formwork for cast-in-place construction, particularly around windows and doors;
- As ceiling boards for casting hempcrete in roof assemblies;
- As permanent internal wall boards for continuous plaster detailing.

There have been efforts made to recycle wood waste into WWCBs, and it has been concluded that up to 30% of wood waste could be used in WWCBs without their properties decreasing [10]. Since WWCBs have continued to gain popularity, there is also a focus on the recycling of WWCBs. Troldtekt offers composting of WWCBs, which helps to neutralize the acids produced during composting [11]. However, this approach leads to material becoming unrecoverable. A bio-based fiber and cementitious binder material was developed by Argalis et al., with the use of WWCB manufacturing waste as the binder material, and subjected to reactivation [11]. It has been calculated that through the introduction of construction and demolition wood waste into cement-bonded particle boards, the global warming potential can be reduced from 733 kg CO_2eq_ to 657 kg CO_2eq_ [12]. Finally, Yilmaz et al. proposed using wood-wool particle board waste as a material for Portland cement production, where it can contribute as fuel, as well as a source of clinker compounds [13].

A high water content is relevant to biocomposites produced with mineral binders, which may possess several latent problems [14,15]. Therefore, solving this in the drying process and lowering the moisture content in biocomposites after production may lead to their safe and sustainable use. Moisture-related risks are the most critical and may be associated with the use of such materials, such as infestation, biological degradation, the presence of moisture, and structural degradation [16]. The initial moisture content and surrounding climate conditions strictly define these parameters. The hydro-thermal properties of hempcrete, such as its sorption/desorption curves, are highly related to environmental moisture, which can vary from 20 to 90% [17]. The behavior of hempcrete walls under realistic conditions revealed that risks of mold growth are subject to both the initial moisture content after production and that after long-term vapor pressure and relative humidity fluctuations [18,19]. The time required to dry specific biocomposites is a question to address before the production of biocomposites to understand the safe use of these materials.

This research investigates the utilization of recycled WWCBs as secondary aggregate to produce cement-bonded biocomposites. The effects of the mixture design and variations in the compaction were evaluated, and the improvement in the mixture’s composition through the addition of hemp shives was investigated. The drying characteristics of the produced biocomposites were measured and described.

## 2. Materials and Methods

### 2.1. Cement-Bonded Particle Board Waste

**Figure 1:**
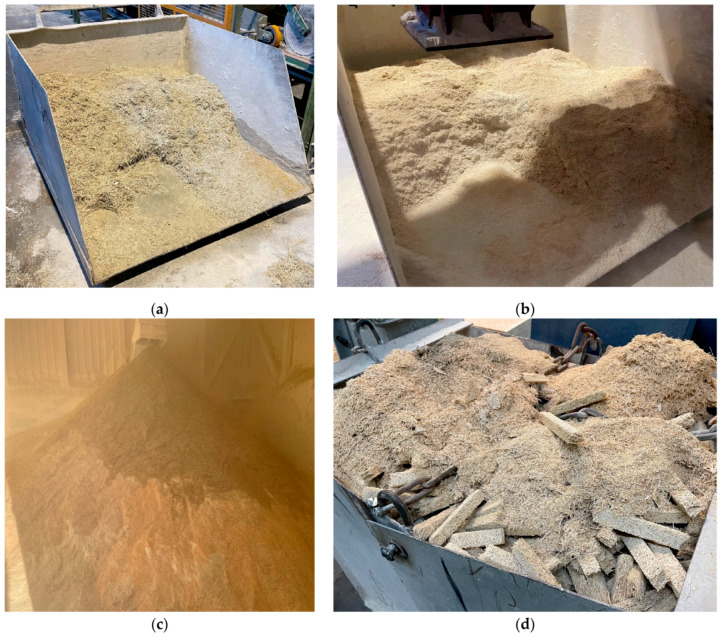
By-products generated during the production of wood-wool cement boards. (**a**) Production line tailings; (**b**) unmolding scraps; (**c**) sanding/grinding dust; (**d**) WWCB scraps from production.

The production process of WWCBs involves shredded wood fibers called wood wool, which are mixed with Portland cement and water in predetermined proportions. Once cured, the WWCBs undergo demolding and finishing processes such as sanding or cutting to achieve the desired dimensions and surface smoothness. During the production of WWCBs, several types of residue streams are generated at different stages of the process, such as excess mixture leftovers, trimmed material, demolding scraps, sawing and sanding dust, and WWCBs treatment scraps. The by-products generated during the production of WWCB by Cewood Ltd. (Jaunlaicene Aluksne, Latvia) factory are given in Figure 1.

**Figure 2:**
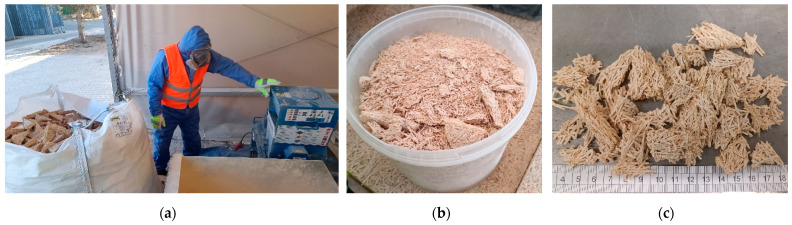
(**a**) Crushing process of production scraps with small-scale hammer mill at Cewood Ltd. Manufacturing plant; (**b**) processed scraps; (**c**) remains larger than 10 mm.

In this research, WWCB scraps (board scraps, BSs) were recycled by processing with a jaw crusher and hammer mill to produce a secondary raw material used as an aggregate fraction to produce lightweight biocomposites. A semi-industrial debris crusher, SIMA TRITON 400 V, with a 10 mm sieve, was used to process WWCB scraps (Figure 2). Triangular scraps from the hexagon WWCB production line were subjected to crushing. Particles < 10 mm were separated for further application.

**Figure 3:**
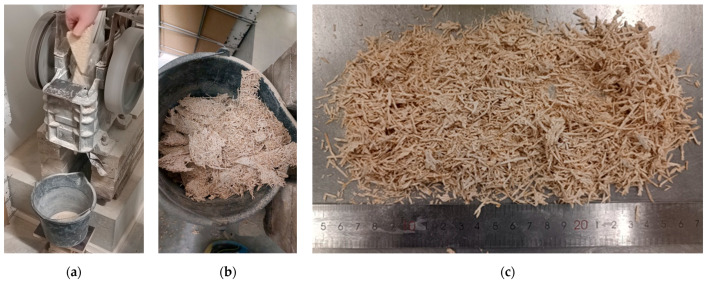
(**a**) Crushing of production scraps in a jaw crusher at the RTU laboratory; (**b**) material crushed with jaw crusher; (**c**) fraction < 10 mm.

WWCB scraps were also processed with a laboratory jaw crusher available at the RTU laboratory (Figure 3). A 3 kW powered jaw crusher with a feed opening of 50 × 120 mm and a discharging hole setting of 10 × 120 mm was used. Repeated crushing was performed to achieve the sought parameters of the recycled aggregate.

Hemp shives (HSs) with fraction sizes from 2 to 40 mm were used as a lightweight aggregate to reduce the density of the biocomposites. The bulk density of HSs can range from 96 to 158 kg/m^3^, depending on the compaction quality. HSs have been described in previous articles published by the authors [20,21].

**Figure 4:**
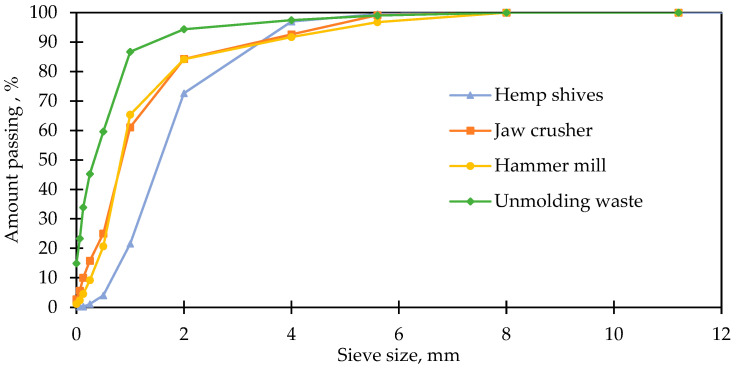
Particle size distribution of recycled WWCB and hemp shives.

The particle size distributions of the treated BSs and hemp shives are given in Figure 4. It was detected that both the hammer mill and jaw crusher give similar recycled aggregate particle size distributions after the treatment of scrap boards. The production line unmolding waste has a similar grading to crushed aggregates, while it contains a much larger fraction of sanding dust (below 0.063 mm) of 23%. The hemp shives used in this research have the largest particles compared to treated BSs. Particles up to 4 mm are common in hemp shive aggregates.

### 2.2. Cement

Commercial Portland limestone cement CEM II/A-LL 42,5N (Schwenk Ltd., Broceni, Latvia) was used as a binder (OPC). Its composition includes Portland cement clinker, gypsum or anhydrite, and lime additives. The total amount of additives was 6–20%, complying with the EN 197-1 standard [22]. The early compressive strength (2 d) was >10.0 MPa, and the standard 28 d strength was >42.5 MPa.

### 2.3. Mixture Compositions

Treated BSs were examined as an aggregate to produce biocomposites. The effects of the binder-to-aggregate ratio and compaction factor were examined and evaluated. The first series of biocomposites were prepared with different OPC-to-BS ratios (Table 1). The OPC-to-BS ratios were 1:2, 1:3, and 1:4. The water–cement ratio increased with the increase in BS content in the mixture composition. The 1:2 mixture had a W/C of 1.1, while in the 1:3 mixture, this increased to 1.7, and in the 1:4 mixture, the W/C increased to 2.0. This increase is associated with BSs’ attraction to water. In previous research, the water-to-bioaggregate ratio was above 1:1 [23,24,25].

The second series involved the effects of biocomposite mixture compaction on the properties of biocomposites (Table 2). The OPC and BS ratios were set as 1:2 to ensure proper binding and self-supporting properties in comparison to lower OPC-to-BS ratios. The W/B ratio ranged from 1.0 to 1.1, basically depending on the volume of the biocomposite mixed in a single batch. Three mixture compositions with the same OPC-to-BS ratios were prepared, and the compaction of the fresh material was different for each mixture. Compaction was performed with the application of additional pressure with a hand trowel. No further mechanical pressing was applied. This simulated the production of BS-based biocomposites in a construction site, where the material is cast in molds. The dependence on compaction could be used to describe the possible deviation of the biocomposite’s properties. The total amount of fresh material that can be consolidated in the formwork, which would affect the final density and properties of the biocomposite, was 550 to 640 kg/m^3^.

To reduce the densities of the biocomposites, which in the first two series were limited by the properties of the aggregate derived from BSs, partial BS substitution by hemp shives (HSs) was evaluated. Previous research has shown that the addition of HSs allows for obtaining biocomposites with a density of 200 to 400 kg/m^3^ [20]. The replacement of 25%, 50%, and 75% by weight of BSs with HSs was assumed (Table 3). The previously derived results for hemp shive size prove that while smaller hemp particles favor a greater interface, increases in hemp shive size lead to greater porosity. This resulted in lower thermal conductivity, lighter hempcrete, and, hence, higher thermal capacitance [26]. The optimum thermal performance was obtained with hemp particles under 3 mm wide and shorter than 10 mm. The OPC-to-aggregate ratio remained the same, at 1:2 by weight. The bulk density of HSs is significantly lower than that of BSs (100 kg/m^3^ for HSs compared to 300 kg/m^3^ for BSs), which is why the total amount of raw materials per 1 m^3^ of biocomposite is reduced. A reduction in the OPC occurred per 1 m^3^ of the biocomposite, as the replacement of BSs with HSs increased the total volume of the mixture. OPC compensation was not included in the mixed design calculations.

### 2.4. Mixing Procedure

**Figure 5:**
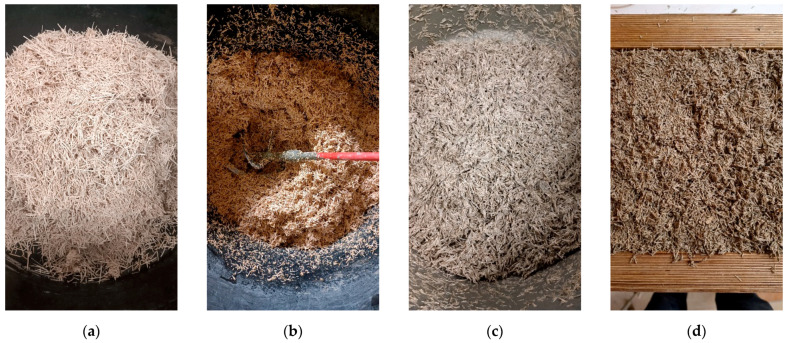
Mixing procedure of biocomposites: (**a**) crushed scrap aggregate; (**b**) pre-wetting of aggregates; (**c**) binder incorporation in the mixture; (**d**) casting of the biocomposite.

All dry components were weighed, but before mixing (Figure 5a), the aggregates were mixed with the mixing water to wet the surface for 2 min (Figure 5b). A single-shaft electrical mixer, RubiMix, was used for mixing. Then, OPC was added, and the mixture was homogenized for 2 min (Figure 5c). The mixture was then cast in prepared molds (Figure 5d). Plate specimens with dimensions of 35 × 35 × 5 to 35 × 35 × 10 cm were prepared for further testing. To obtain homogeneity in the prepared samples, the exact weights of the mixed material were placed in the molds.

### 2.5. Test Methods

Samples were demolded after 7 days of curing in molds. Then, they were air-dried at 22 ± 2 °C and RH 50 ± 5% until the 28th day, when they were placed in a climate chamber for conditioning at 45 °C until a constant mass was achieved. Then, bulk density was determined, and thermal conductivity was measured with a LaserComp FOX600 heat flow meter; according to the standard EN 12667 guidelines; the settings of the test were 0 °C for the upper plate and 20 °C for the lower plate [27]. After the test, biocomposite samples were cut for the flexural and compressive strength tests. Compressive strength tests were performed until 10 and 20% relative deformations in the casting direction (according to the LVS EN 826) using a Zwick Z100 universal testing system (ZwickRoell, Kennesaw, GA, USA) with a testing speed of 0.5 mm/min [28]. Flexural strength was tested until critical load was achieved in a direction perpendicular to casting at a speed of 0.5 mm/min; 60 × 30 × 200 mm samples were tested.

The samples were characterized with a digital microscope (Veho HDMI Dual Vision Digital), assessing their structure and appearance. The drying characteristics of the biocomposites were measured for prismatic specimens; we employed samples with a surface area of 6 × 18 cm and thickness of 2, 4, and 6 cm. Ling Pigeon S265 temperature and humidity loggers with AM2301 sensors were used. The humidity sensors were wrapped in a plastic fiber cloth to avoid excess moisture damage. The drying environment was 22° ± 2 °C, and the RH was 30% ± 5.

## 3. Results

The physical properties of the obtained biocomposites are given in Table 4. The bulk densities of the biocomposites increased as the cement-to-aggregate ratio increased. An increase in the OPC-to-BS ratio from 1:2 to 1:4 decreased the bulk density of the biocomposites from 474 to 391 kg/m^3^. The decrease in bulk density reduced thermal conductivity from 0.082 W/(mK) to 0.070 kg/m^3^. This gradual decrease is associated with dense cement particle reduction in the composition. The second series’ results indicate that compaction from 416 to 511 kg/m^3^ can be reached by hand. During the mixing, BSs are scattered and stirred up such that it is easy to reach different degrees of compaction. The thermal conductivity was related to the bulk density. It was in the range of 0.077 to 0.084 W/(mK). This correlates well with the results of the first series of samples. The third series with HS incorporation reduced bulk density significantly. Even a 25% incorporation of HSs reduced bulk density to 329 kg/m^3^. Bulk density reduced to 197 kg/m^3^ with 75% BS substitution with HSs (H75), and a decrease in thermal conductivity was achieved with such an approach. An effective value of 0.054 W/(mK) can be reached. However, little reduction was observed for the H50 and H75 mixtures.

**Figure 6:**
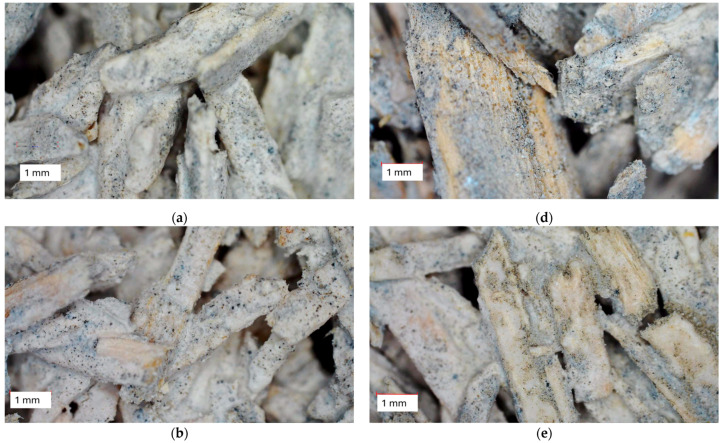

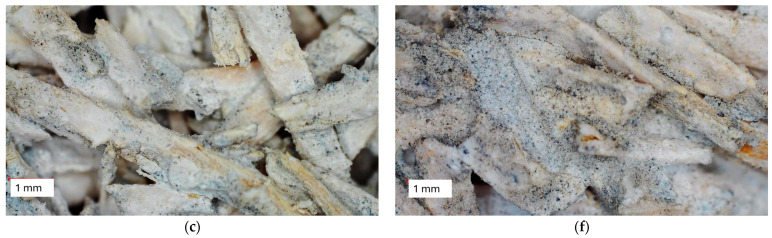
Macrostructures of the biocomposites. (**a**) Composition I; (**b**) composition II; (**c**) composition III, (**d**) composition H25; (**e**) composition Ia; (**f**) composition Ib.

The macrostructures of the biocomposites are given in Figure 6. The BS aggregates were completely covered with cement particles, and only for mixture H25 could large-size hemp shives be identified separately. Hemp shives have a larger particle size distribution than BSs; therefore, it is much easier to identify individual hemp shives. The presence of large particles with low density reduces the downgrading effect of the OPC, with increases in thermal conductivity. Mixture compositions containing only BSs and OPC have similar microstructures. More compacted biocomposites (Ib) seem to have lower porosity, as fewer voids between particles can be observed. The change in OPC content in the mixture composition had a minor effect on the microstructure observed by microscope.

**Figure 7:**
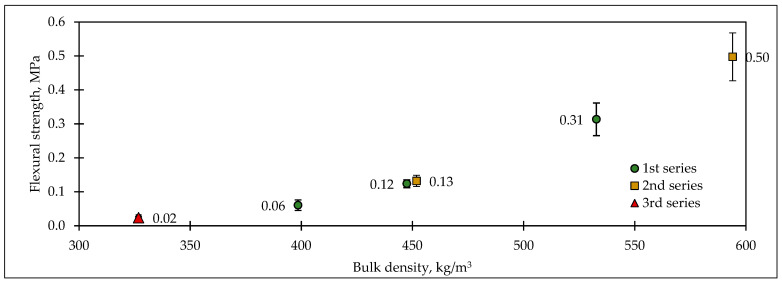
Flexural strengths of the biocomposites.

The flexural strength is given in Figure 7. The results show that the biocomposite with the highest density provided the highest compressive strength—0.5 MPa. The decrease in density reduced flexural strength significantly. The highest amount of OPC in the biocomposite led to a compressive strength of 0.31 MPa. The reduction in OPC led to a decrease in strength to 0.06 MPa. However, the lowest strength was reached by the mixture with HSs. Only H25 could be tested, and its strength value was as low as 0.02 MPa. Different compactions of biocomposites could change the flexural strength from 0.13 to 0.50 MPa.

**Figure 8:**
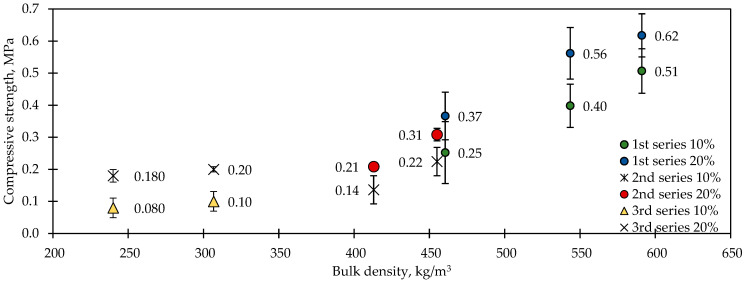
Compressive strengths of the biocomposites.

Compressive strength results are given in Figure 8. The compressive strength was affected most by the cement content and the compaction of the biocomposites. The most compacted biocomposite had a compressive strength of 0.5 MPa at 10% deformation and 0.6 MPa at 20% deformation. The strength was reduced due to the decrease in OPC content to 0.1 MPa at 10% deformation and 0.2 MPa at 20% deformation. The loosely compacted biocomposites showed a decrease in strength to 0.14 MPa at 10% deformation and to 0.21 MPa at 20% deformation. The incorporation of HSs reduced the compressive strength even more. The H25 composition showed a compressive strength of 0.10 MPa at 10% deformation and 0.20 MPa at 20% deformation; similar results were obtained for H50.

**Figure 9:**
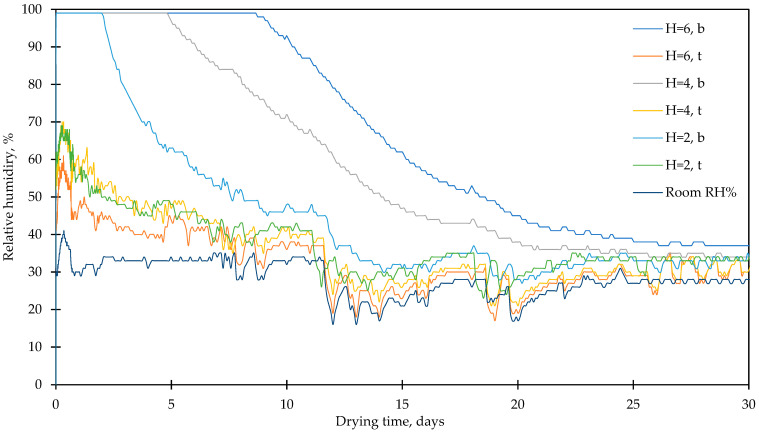
Drying characteristics of the biocomposites.

The drying kinetics of biocomposite mixture composition I are given in Figure 9. The results indicate that the drying speed is dependent on the thickness of the material. Here, 2 cm thick biocomposites started drying after 2 days, while samples that were 4 cm thick started drying after 5 days, and 6 cm thick materials started to dry after 9 days. After 12 days, the RH in the whole sample was below the level of 40% achieved by the 2 cm sample. For the 4 cm sample, this level was reached after 19 days. For the 6 cm sample, it was reached after 24 days. On the surfaces, the RH reached 60% for 2 cm thick samples and 70% for 4 and 6 cm thick samples. The moisture content on the surface reduced to the surrounding RH after 4 days for the 2 cm thick samples and after 7 days for the 4 and 6 cm thick samples.

The results of drying characteristics are compiled in Table 5. They indicate that, in total, 130 kg/m^3^ of water needs to evaporate to reach a level of 40% RH inside the biocomposite, which means the total amount of free water in the composite is between 27 and 28 wt. %. The thicker samples showed a much slower drying rate compared to the 2 cm sample (5.4 kg_water_/day for 6 cm samples and 11.2 kg_water_/day for 2 cm samples). However, when calculated per free surface area from which moisture can be released, a higher drying rate was attained by the 6 cm sample—32.5 kg_water_/m^2^/day, compared to 22.3 kg_water_/m^2^/day for the 2 cm thick sample.

## 4. Discussion

Bio-based cement composites have gained popularity around the world due to their versatility and properties, as provided by the two main raw materials—wood fibers, which are a renewable resource with high mechanical (predominantly tensile) properties, and Portland cement, which binds together individual particles, gives compressive strength, and improves fire resistance in the composite [29,30]. The utilization of waste wood materials, both as wood powders and wood fibers, offers a more environmentally friendly alternative for recycling industrial debris compared to current waste management practices. This approach also conserves natural resources and reduces the CO_2_ emissions associated with producing raw materials for manufacturing these mortars [31]. The production process of wood-wool cement particle boards (WWCBs) is associated with a small fraction of prepared material ending up as a residue, which, together with damaged and end-of-life boards, needs to be recycled and reused. The crushing of such WWCBs can offer secondary aggregates for the development of new biocomposites. In this study, two industrially feasible methods were used to crush the material, and the aggregate grading outcome was similar. This must be associated with the brittle nature of the dry WWCBs, which were subjected to processing. The main difference is associated with the crushing efficiency and the contents of fines. The crushing efficiency is more related to the machinery’s working principles and the size of the initial material. The content of fines, which was significantly higher for production residues, is associated with the source of the residue—whether it comes from the production process or from scrap boards.

The production process of new biocomposites follows an easy and reproducible method that is feasible to apply both in a factory and at a construction site. This research has examined the properties of biocomposites obtained from recycled WWCBs, and the mixture proportions and compaction factor of the biocomposites were evaluated. In the literature, there are reports on the production of hemp-based biocomposites with cement-to-filler ratios from 1:1 to 1:4 [23,24,25,32]. In previous studies utilizing WWCBs in the production of new composites, the bulk density achieved by such materials was 430–617 kg/m^3^, while the thermal conductivity was from 0.083 to 0.117 W/mK [11]. This study shows the possibility of obtaining lower-density biocomposites with recycled WWCB aggregates. The thermal conductivity value was decreased to as low as 0.054 to 0.055 W/(mK) with 50 and 75 wt. % HS incorporation, and such an approach allowed us to reach a high efficiency compared to the bio-based insulation materials used in today’s civil engineering. However, conventional thermal insulation materials have a thermal conductivity of 0.025 to 0.046 W/(m·K) [33]. Depending on the OPC content and the compaction force applied during the production, the lowest density achieved with recycled WWCB aggregate was 391 kg/m^3^. The compaction factor had a significant effect on the density of the obtained biocomposites. The same mixture composition can be compacted with a final bulk density of 416 to 511 kg/m^3^, which represents a significant deviation from the expected results. An increase in the OPC-to-BS ratio led to a decrease in density from 474 to 391 kg/m^3^, while a significant decrease in strength properties was also determined.

In previous studies, hemp shives proved to be one of the best aggregates for the production of high-efficiency hempcrete [34,35,36,37]. Here, new biocomposites consisting of hemp shives and recycled WWCBs were proposed and examined for the first time. The results are sustainable, as the bulk density decreased from 474 to 197 kg/m^3^, and even more importantly, the thermal conductivity reached the same value as that of bulk hemp shives (0.054 W/(mK)). In such a way, low-strength self-bearing biocomposites with natural hemp shives and recycled WWCB aggregates can be efficiently utilized. The recycled WWCB aggregates can increase fungal resistance, which is important as spruce timber is used in the production of WWCBs, and it is partly covered with cement particles. Fire resistance can be hypothetically improved in accordance with the findings of previous papers dedicated to the investigation of hemp-shive-based biocomposite fungal and fire resistance. At the same time, both properties must be investigated in further research [38].

The mechanical performance of the biocomposites was closely related to the amount of binder used in the preparation of the samples, as well as the density of the produced materials. The hydrated cement particles, which remain in the crushed aggregate, increase the density of the filler, lowering thermal efficiency and increasing the weight of the material. Extra water could also be attracted during the production since crushed fine hydrated cement particles could absorb water. The incorporation of hemp shives reduced the density significantly, while the strength of the biocomposite also decreased. Compared to commercial wood-wool cement composite boards, the material density of which varies from 350 to 520 kg/m^3^, those obtained in this research are similar, while the strength of such primary material boards ranges from 0.3 to 0.6 MPa. The compressive strength of the biocomposites produced from recycled material is similar, while the bending strengths of commercial boards are much higher—from 0.7 to 2.8 MPa. This is associated with the presence of the long wood-wool strands of which the commercial boards are made.

The drying of biocomposites prepared with mineral binder is a topical issue in civil engineering, as a high initial water content reduces the thermal efficiency and insulation capabilities of the biocomposite, as well as the risk of fungal development in moist biocomposites. The initial water content in biocomposites is important for cement hydration, as biological fiber, such as HSs and BSs, can increase the degree of hydration by about 9–9.5% for OPC mortar, and different minerals are formed during curing [39]. It was established that around 130 kg/m^3^ of free water should be evaporated from the prepared biocomposites. In relatively dry conditions, the complete drying can take from 12 to 24 days, depending on the thickness of the material. In this research, the drying rate varied from 5.4 to 11.2 kg of free water per day, while the efficient drying rate at the free material’s surface was from 22.3 to 32.5 kg of free water per square meter per day. This gives valuable information about drying characteristics and durations, which is of great relevance to the application of such biocomposites. Further, hemp–lime composite building materials have also been proven to have an excellent moisture-buffering capacity, but their prolonged high moisture content can lead to fungal growth in and damage to the biocomposite [25].

## 5. Conclusions

The present research has investigated the recycling possibilities of wood-wool cement boards (WWCBs). Crushing damaged or recycled WWCBs creates bio-based aggregates that can be used for the further development of biocomposites. The new biocomposites, made with Portland-cement-to-binder ratios of 1:2 to 1:4, have a density of 391 to 511 kg/m^3^. The cement content affects the strength characteristics and thermal conductivity. The lowest thermal conductivity obtained was 0.070 W/(mK), which was limited by the properties of aggregates, as crushed WWCBs contain wood wool and cement particles. To improve the thermal performance of the biocomposites, hemp shives were introduced into the composition, substituting the aggregate at 25 to 75 wt. %. As a result, the bulk density was decreased to 197 kg/m^3^, and the thermal conductivity was 0.054 W/(mK). At the same time, the strength of such a material is limited, which can be attributed to being a self-bearing insulation material. The most critical finding was that the drying of such composites could take up to 24 days for a 6 cm thick sample, and a drying rate of 22.3 to 32.5 kg of water per m^2^ per day was measured. This research offers a feasible approach to recycling WWCBs into new, efficient bio-based insulation materials. The future challenges of recycled aggregate biocomposite application are related to the long-term durability of the biocomposites in real-world applications and the further refinement of the recycling process to reduce cement contamination.

## Figures and Tables

**Table 1:** The biocomposites were made with CEMII and a wood-wool cement composite board scrap filler in different proportions (first series).

Component	Mixture Composition, kg/m^3^		
	I	II	III
BSs	300	300	300
OPC	150	100	75
H_2_O	165	160	150
	Mixture mass ratios		
BSs	2	3	4
OPC	1	1	1
H_2_O	0.5	0.33	0.25
*W/C*	1.1	1.7	2.0

**Table 2:** A mixed-composition biocomposite was made with OPC and BSs with different compactions (second series).

Component	Mixture Composition, kg/m^3^		
	Ia	I	Ib
BSs	270	300	320
OPC	135	150	160
H_2_O	144	166	160
	Mixture mass ratios		
BSs	2	2	2
OPC	1	1	1
*OPC/BSs*	0.5	0.5	0.5
*W/C*	1.1	1.1	1.0

**Table 3:** Mixture composition of biocomposites with hemp shives (third series).

Component	Mixture Composition, kg/m^3^			
	H0	H25	H50	H75
BSs	300	160	80	32
Hemp shives (HSs)	0	55	80	96
OPC	150	107	80	64
H_2_O	166	194	156	155
	Mixture mass ratios			
HS/BS	0	0.33	1	3
OPC/aggregate	0.50	0.50	0.50	0.50
W/C	1.1	1.8	2.0	2.4

**Table 4:** Physical properties of the biocomposites.

Property	I	II	III	Ia	Ib	H25	H50	H75
Bulk density, kg/m^3^	474	415	391	416	511	329	240	197
Thermal conductivity, W/(mK)	0.082	0.077	0.070	0.077	0.084	0.064	0.055	0.054

**Table 5:** Analysis of drying characteristics of the biocomposite.

Sample Thickness, cm	Moisture Content, %	Free Moisture Evaporated kg_water_/m^3^	Drying Time, d	Drying Rate, kg_water_/Day	kg_water_/m^2^/Day
6	27	130	24	5.4	32.5
4	27	130	19	6.8	27.3
2	28	134	12	11.2	22.3

## Data Availability

The original contributions presented in the study are included in the article, further inquiries can be directed to the corresponding author.
